# Telepathology in emerging countries pilot project between Italy and Egypt

**DOI:** 10.1186/1746-1596-3-S1-S2

**Published:** 2008-07-15

**Authors:** Essam Ayad, Francesco Sicurello

**Affiliations:** 1 Departament of Pathology. Faculty of Medicine, Cairo University & Italian Hospital in Cairo, Egypt; 2 @ITIM-Italian Association of Telemedicine and Medical Informatics, University of Milano Bicocca, Dept. of Informatics, Systemistic and Communication, Lombardia Region – Health Directorate, Italy

## Abstract

Pathological examination includes gross & microscopic examinations at different magnification. Through the steps of examination, we obtain many images that can be used for telepathology. Telepathology is the practice of pathology at a distance, viewing images on a monitor rather than directly through a light microscope. It can be used for primary diagnosis, second opinion, quality assurance and distance learning. Telepathology is classified into Static, Dynamic, Hybrid and Whole Slide Imaging (WSI). We have a successful experience in Egypt in applying the static & dynamic techniques in a pilot project between the Italian Hospital in Cairo (NPO) and the Civico Hospital in Palermo. This project began in 2003 and continued till now. From the second year 2004, Ospedale S. Giovanni e Paolo Hospital in Venice, Charing Cross Hospital in London and the University of Pittsburgh Medical Center Health System (UPMC) in the USA participated actively in our project. During the past five years we consulted on many problematic pathological cases with these different specialized pathological centers in Italy, UK & USA. In addition to the highly specialized scientific value of consulting on the cases and exchanging knowledge, we saved a lot of time and money and succeeded in providing our patients with a better medical service.

We are now in the process of establishing a Digital Telepathology Center (DTC) in the pathology department, Cairo University, using the latest technique of telepathology which is Whole Slide Imaging (WSI). We believe that it will help us to improve and extend diagnosis for our difficult pathological cases and will facilitate increased E-learning opportunities for staff and students both in Egypt and in the longer term in the wider Eastern Mediterranean.

## Introduction

Telepathology is the practice of pathology at a distance, viewing images on a monitor rather than directly through a light microscope. In today's health care system, there are many uses of telepathology. For example, to "provide urgent services at sites either without a pathologist or sites with a pathologist requiring additional professional back-up." Also, telepathology can "provide immediate access to sub-specialty pathology consultants." (Telemedicine: Theory and Practice). In general, telepathology techniques are classified into Static, Dynamic, Hybrid and Whole Slide Imaging (WSI).

## Materials and methods

**Figure 1:**
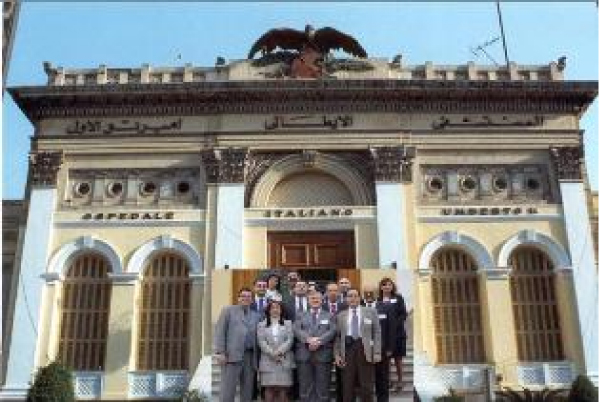
The Italian-Egyptian Telemedicine Committee (Picture taken at the Italian hospital in Cairo, 2003).

We have a successful experience in Egypt in the application of the static and dynamic techniques of telepathology through a pilot project between the Italian Hospital in Cairo (NPO) and Civico Hospital in Palermo [1]. This project began in 2003 by initiating the idea of the cooperation between CIVICO hospital in Palermo and the Italian hospital in Cairo, followed by the signing of a protocol of cooperation between both hospitals (Figure 1). The project is divided into four phases: Telepathology, Tele-echocardiography, Tele-radiology & Tele-Endoscopy. The first phase (Telepathology) is now practically mature. The next phases of our project will be: Tele-echocardiography, Tele-radiology & Tele-endoscopy.

For the First phase (Telepathology), these steps were taken: evaluation of the costs and securing the required funding; searching for the best instruments in the Egyptian market with the required functionality and certifications; downloading, and using in Cairo, the same software as used in Palermo; and the beginning of the connection. In addition during the second year (2004), Ospedale S. Giovanni e Paolo Hospital in Venice, Charing Cross Hospital in London and University of Pittsburgh Medical Center Health System (UPMC) in USA joined as active participants in the telepathology project.

**Figure 2:**
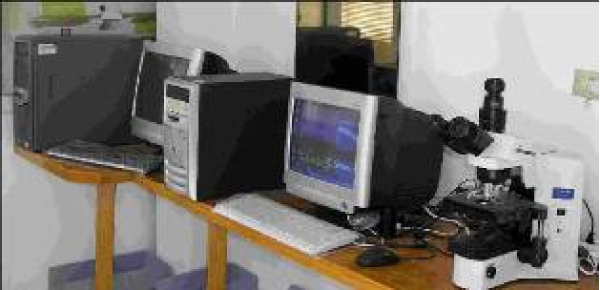
The Telepathology Unit in Italian hospital in Cairo.

The essential needs for our complete telepathology unit were straightforward: a suitable place, a server with a high-speed internet connection, a full computer system (computer, scanner & printer, a binocular microscope, a digital camera for gross pathology, a digital camera connected to the microscope to capture images from the microscope, a video-camera connected to the computer for video-conferences, and suitable software for image processing and image transfer via the internet (Figure 2) [2].

The benefits we expected and have achieved from the introduction of this telepathology unit are clear: better medical service, more distributed specialization, savings in time and money, increased knowledge exchange provides strong basis for improved teaching and learning practices. This unit has also provided its services to any pathology department in the Egyptian universities or any research centre.

## Results

Over the past 5 years we consulted alot of problematic pathological cases with these different specialized pathological centres in Italy, UK & USA. Beside the highly specialized scientific value of consulting the cases and exchanging knowledge, we saved a lot of time and money and offered our patients a better medical service [3,4].

## Conclusion

We concluded from our experience that telepathology is a very useful and applicable tool for additional consulting on difficult pathological cases. It has significantly increased knowledge exchange and thereby ensured our patients a better medical service, while simultaneously saving a lot of time and money over the previous practices. In view of this success and the networks established we now are in the process of establishing a Digital Telepathology Center (DTC) in the pathology department, Cairo University. This will include use of the latest technique of telepathology, which is Whole Slide Imaging (WSI). We believe that it will help us to improve and extend diagnosis for our difficult pathological cases and that the discussion of our cases will facilitate and ensure increased E-learning opportunities for staff and students both in Egypt and in the longer term in the wider Eastern Mediterranean.
